# Chronic Obstructive Pulmonary Disease: A Growing but Neglected Global Epidemic

**DOI:** 10.1371/journal.pmed.0040112

**Published:** 2007-05-15

**Authors:** Peter J Barnes

## Abstract

Peter Barnes argues that COPD is a major and increasing global health epidemic that has received insufficient attention from the health-care profession, governments, and the pharmaceutical industry.

Chronic obstructive pulmonary disease (COPD) is a major and increasing global health epidemic that has received insufficient attention from the health-care profession, governments, and the pharmaceutical industry. Urgent action is now required to recognise the disease, predicted to soon become one of the major causes of death and disability, and to develop more effective prevention and treatment strategies.

## What Is COPD?

COPD is described by the Global Initiative for Chronic Obstructive Lung Disease as “a preventable and treatable disease…characterized by airflow limitation that is not fully reversible. The airflow limitation is usually progressive and associated with an abnormal inflammatory response of the lung to noxious particles or gases” [1]. This progressive and relentless loss of lung function is caused by emphysema due to destruction of lung parenchyma and by narrowing of small airways as a result of chronic inflammation and fibrosis and loss of elastic recoil. This results in progressive airflow limitation, air trapping, and progressive shortness of breath on exertion.

## The Size of the Problem

The Global Burden of Disease studies ranked COPD as the sixth commonest cause of death worldwide in 1990, and it was predicted to become the third commonest cause by 2020 [2]. A more recent projection from the World Health Organization predicts that it will rise from its current ranking as fifth commonest cause of death to be the fourth commonest by 2030, behind only ischemic heart disease, cerebrovascular disease, and HIV/AIDS [3]. The increase is predicted to be greater in developing countries than affluent countries. Indeed, COPD is the only common cause of death in the United States that has increased over the last 40 years, in sharp contrast to the reduction in cardiovascular and infectious diseases. Even more importantly, COPD is an increasing cause of chronic disability and is predicted to become the fifth most common cause of chronic disability worldwide by 2020 [2]. The prevalence of COPD is approximately 10% in the general population throughout the world [4,5]. Many patients with COPD remain undiagnosed even when the disease causes severe disability [6].

Consultation rates for COPD in general practice in the United Kingdom now exceed those of ischemic heart disease by 2-4-fold, and COPD is one of the commonest causes of hospital admission in the UK [7]. COPD is also one of the commonest reasons for time lost from work, placing an enormous and increasing economic burden on society. This has resulted in major health-care expenditure that now exceeds the costs of asthma by over 3-fold [6]. The reasons for the global increase in COPD include continuing cigarette smoking amongst men, with increasing smoking amongst women, and the longer survival of populations; these changes are particularly relevant in developing countries.

## The Neglect of COPD

Despite growing recognition as an important international health problem, COPD has suffered neglect from clinicians, researchers, and the pharmaceutical industry [8]. This is largely because COPD is viewed as self-inflicted (by smoking) and also because the underlying disease process is generally perceived to be irreversible. Consequently, there is a fundamental lack of knowledge about the cellular, molecular, and genetic causes of COPD. Existing therapies for COPD are inadequate and none have been shown to slow the relentless progression of the disease. In terms of research funding, COPD has been relatively neglected among common diseases, with little investment in research into its underlying cellular and molecular mechanisms. COPD is now recognised to have the greatest socioeconomic inequality of any common disease and is commonly a disease of the poor, suggesting that there are environmental factors other than smoking contributing to the disease. Indeed, over 10% of patients with a clinical diagnosis of COPD are non-smokers, and this proportion is much higher amongst women in developing countries such as India, where exposure to biomass fuels in an enclosed space is an important cause of COPD [9].

## Natural History

The classical epidemiological studies of Fletcher and Peto demonstrated that death and disability from COPD were related to an accelerated decline in lung function with time, with a loss of 50–100 ml in forced expiratory volume in one second (FEV_1_) per year, compared to the normal loss of <30 ml per year. Only about 10% of smokers were thought to develop COPD, suggesting that genetic or other environmental factors may play an important role in susceptibility. Apart from FEV_1_, exercise capacity, exacerbation frequency, and systemic features all indicate a poor prognosis [10]. A more recent epidemiological study with a longer follow-up (25 years) suggests that over 25% of smokers may develop COPD, with no difference in susceptibility between men and women [11]. The implication of this study is that the prevalence of COPD amongst smokers is likely to rise even more as people survive longer.

## Inadequate Current Therapy

Smoking cessation strategies have a poor success rate, with the most effective approaches yielding a quit rate of only about 15% [12]. In any case, stopping smoking as the disease becomes more severe has little impact on its progression [13], and several studies have now shown that smoking cessation fails to reverse the chronic airway inflammation [14]. This stresses the need for more effective anti-smoking strategies, including new and more effective drugs for nicotine addiction, and earlier intervention. Anti-inflammatory drugs, which are used so successfully to manage asthma, have few clear beneficial effects in COPD and there is a very poor response to corticosteroids due to an active resistance against the anti-inflammatory actions of steroids [15]. Bronchodilators, which are the mainstay of current drug therapy, do not significantly affect the underlying disease process and therefore do not slow disease progression towards respiratory failure and death. New treatments for COPD, including the development of new classes of drugs, are urgently needed [16], and although progress has recently been made in understanding the molecular, cellular, and genetic mechanisms involved in COPD, far more research is required in this area [17]. This may lead to a better understanding of the mechanisms for disease progression and to the development of effective therapies in the future.

## What Are the Solutions?

It is clearly necessary to increase awareness of COPD amongst health-care professionals, the general public, and governments. General practitioners must be educated about how to recognize COPD and institute the most appropriate therapy. The Global Initiative for Chronic Obstructive Lung Disease plays an important role in raising awareness of COPD amongst health-care professionals and has formulated updated evidence-based guidelines for diagnosis, therapy, and prevention [1]. Stopping smoking early is very important, particularly for those in the early stages of the disease, in whom smoking cessation has a clear benefit and reduces mortality [18]. More research is needed into the underlying disease mechanisms, to identify the genetics of susceptibility and to identify new targets for treatment. It is increasingly recognised that there is heterogeneity in the disease and more careful phenotyping is required in the future to elucidate disease mechanisms and optimise novel therapies. This will require much more investment from funding bodies and governments. The attitude that smoking-related lung diseases are self-induced and therefore less worthy of attention needs to be changed; this attitude does not appear to apply to the same extent to ischemic heart disease, diabetes, or obesity. Non-smoking causes of COPD will require far more attention in the future and the genetic and environmental cofactors that interact with COPD need to be more carefully explored. Far more research needs to be done in developing countries where non-smoking causes of COPD account for a much greater proportion of COPD than in developed countries. The striking socioeconomic disparities in COPD prevalence implicate factors other than smoking, but these factors have so far largely been ignored. In the UK, respiratory disease, which includes COPD, receives less funding in relation to the burden of disease than any other disease area [19]. COPD will undoubtedly place an increasing burden on health resources and this burden is likely to be particularly severe in developing countries. Finally, COPD needs to be more prominently featured in peer-reviewed journals that address global health issues—as recently pointed out by the editors of this journal [20].

## Abbreviations

COPD: chronic obstructive pulmonary disease
FEV_1_: forced expiratory volume in one second
